# Post-discharge complications and hospital readmissions are associated with nutritional risk and malnutrition status in a cohort of Canadian pediatric patients

**DOI:** 10.1186/s12887-024-04941-6

**Published:** 2024-07-23

**Authors:** Joëlle Létourneau, Véronique Bélanger, Valérie Marchand, Dana L. Boctor, Mohsin Rashid, Vishal Avinashi, Véronique Groleau, Schohraya Spahis, Emile Levy, Valérie Marcil

**Affiliations:** 1 Department of Nutrition, Université de Montréal, Research Center CHU Sainte-Justine, 3175 Ch de la Côte-Sainte-Catherine, Room 4.17.006, Montreal, QC H3T 1C5 Canada; 2 Department of Pediatrics, Université de Montréal, CHU Sainte-Justine, Montreal, QC Canada; 3 Alberta Children’s Hospital, University of Calgary, Calgary, AB Canada; 4 IWK Health Center, Dalhousie University, Halifax, NS Canada; 5 Division of Gastroenterology, Hepatology and Nutrition, British Columbia Children’s Hospital, Vancouver, BC Canada

**Keywords:** Child, Hospitalized, Malnutrition, Nutritional Status, Patient Readmission, Risk Assessment

## Abstract

**Background:**

This study constitutes a secondary analysis of a prospective cohort aiming to evaluate the potential correlation between nutritional risk and status at admission with the occurrence of post-discharge complications and hospital readmissions in children receiving care at high resource Centres.

**Methods:**

Data was collected from 5 Canadian tertiary pediatric Centers between 2012 and 2016. Nutritional risk and status were evaluated at hospital admission with validated tools (STRONGkids and Subjective Global Nutrition Assessment [SGNA]) and anthropometric measurements. Thirty days after discharge, occurrence of post-discharge complications and hospital readmission were documented.

**Results:**

A total of 360 participants were included in the study (median age, 6.1 years; median length of stay, 5 days). Following discharge, 24.1% experienced complications and 19.5% were readmitted to the hospital. The odds of experiencing complications were nearly tripled for participants with a high nutritional risk compared to a low risk (OR = 2.85; 95% CI [1.08–7.54]; *P* = 0.035) and those whose caregivers reported having a poor compared to a good appetite (OR = 2.96; 95% CI [1.59–5.50]; *P* < 0.001). According to SGNA, patients identified as malnourished had significantly higher odds of complications (OR, 1.92; 95% CI, 1.15–3.20; *P* = 0.013) and hospital readmission (OR, 1.95; 95% CI, 1.12–3.39; *P* = 0.017) than to those well-nourished.

**Conclusions:**

This study showed that complications and readmission post-discharge are common, and these are more likely to occur in malnourished children compared to their well-nourished counterparts. Enhancing nutritional care during admission, at discharge and in the community may be an area for future outcome optimization.

**Supplementary Information:**

The online version contains supplementary material available at 10.1186/s12887-024-04941-6.

## Background

Nutritional status is a key determinant of a child’s health. Malnutrition or undernutrition is defined as an imbalance between nutrient requirements and intake resulting in cumulative deficits of energy, protein, or micronutrients, which may negatively affect growth, development and other outcomes [1, 2]. In high income countries, a poor nutritional status can be caused by various mechanisms linked to decreased dietary intake, increased nutrient losses, and altered utilization of nutrients [3–7]. These mechanisms are frequently related to an acute or chronic illness of hospitalized children [8]. Thus, understanding the etiology of malnutrition is necessary to implement adequate medical or nutritional interventions.

Nutritional risk screening and complete nutritional status assessment both play an important role in identifying malnutrition and its severity. Given the complexity of malnutrition development and the consequences of inadequate nutritional intake, it has been proposed that the traditional nutritional assessment based on anthropometric measurements is less applicable to hospitalized children with acute and chronic illness [1, 2, 9]. For pediatric patients admitted to intensive care unit or for surgery related to Crohn Disease, a weight-for-age of -1.04 z-score and a mild malnutrition status (BMI-for-age of -1.0 to -2.0 z-score), which represent normal growth according to the WHO definition, have been described as the thresholds at which mortality risk and complication rates increase, respectively [10–12]. As such, early identification of pediatric patients at nutritional risk is key to implementing effective nutrition intervention [13]. Screening tools should be easy to use, quick, cheap, standardized, and validated, thus providing each professional the ability to use them efficiently with minimal training. To detect nutritional risk, various screening tools exist and have been tested in different settings [14, 15]. The Screening Tool for Risk Of impaired Nutritional status and Growth (STRONGkids) is a four-question nutritional risk screening tool developed in the Netherlands. In a nationwide study, children at nutritional risk had a higher prevalence of acute malnutrition, a lower weight-for-height standard deviation score and a prolonged length of stay (LOS) compared to their no-risk counterparts [16]. Similar results were found in large cohorts internationally [16–21]. Children with moderate or high nutritional risk should receive timely nutritional care including a nutritional assessment performed by a registered dietitian since diagnosing malnutrition requires clinical training and expertise. Nutritional assessment consists of evaluating patient’s clinical history, dietary background, anthropometric, and laboratory data. The Subjective Global Nutrition Assessment (SGNA) is a comprehensive nutritional assessment tool covering both phenotypic and etiologic factors linked to nutritional status in children [22, 23]. This tool was adapted from the Subjective Global Assessment (SGA), a widely used questionnaire in clinical and research settings to determine the presence and the degree of malnutrition of adult patients [22]. Studies have validated its accuracy by showing that children classified as malnourished had higher infection rate, increased LOS and higher readmission rate [13, 17, 24–26].

The prevalence of pediatric malnutrition in acute care varies across institutions [27] with a range of 3–50% depending on the population studied and definitions used. Recently, a multi-centred Canadian study reported that 37% of children are malnourished upon hospital admission [17]. Several evidence indicate that pediatric malnutrition defined by growth indicators may be a contributing factor to increased LOS, higher risk of 30-day complications, and mortality [10, 12, 28, 29]. In contrast to these findings, no relation has been found between malnourished children and in-hospital risk of complications and rates of readmission [22, 29]. Aside from the longer LOS and in-hospital complications [17, 22], there is limited data on the impact of malnutrition status after discharge in Canadian pediatric hospitals.

In practice, pediatric malnutrition is often unrecognized and poorly managed [30, 31]. Only 30% of Canadian pediatric healthcare professionals have reported to use of the “malnutrition” coded diagnosis according to the International Statistical Classification of Diseases and Related Health Problems, 10th Revision, Canada (ICD-10-CA) in the discharge summary [31]. The ICD-10-CA coded diagnosis is used by the Canadian Institute for Health Information to determine various health indicators [32, 33]. Among them, the Pediatric Patients Readmitted to Hospital indicator uses, in addition to ICD-10-CA coded diagnosis, other contributing risk factors as covariates such as sex, age group, urgent admission, previous acute care admission within 6 months to determine an adjusted-risk 30-day readmission rate [33, 34]. However, as malnutrition is still under-identified and under-reported in Canada [31], its impact and consequences remain poorly defined.

While the consequences of malnutrition post-discharge are well documented in adults in a high resource setting [35–38], there is a need to assess the outcome of malnourished children 30-day after discharge. This study aims to identify the nutritional factors that influence the occurrence of post-discharge complications and hospital readmissions in pediatric patients.

## Methods

### Study design and protocol

This study is part of a multicenter prospective approach that was conducted in 5 pediatric hospitals across Canada between 2012 and 2016. Centers included Alberta Children's Hospital, CHU Sainte-Justine, IWK Health Centre, Kingston General Hospital, and British Columbia Children’s Hospital. The overall objective of the project was to assess prevalence, causes and consequences of malnutrition in Canadian pediatric hospitals. A first assessment of the cohort focusing inpatient malnutrition prevalence was conducted and published by Belanger et al. [17]. Data collection was completed at admission, every two days throughout hospital stay, upon discharge and 30 days post-discharge using data collection forms designed for the study, which were used uniformly between centers (see Supplementary Materials). Children aged from 1 month to 18 years who were admitted on a medical or surgical ward and had a planned hospital stay of at least 2 days were eligible. Exclusion criteria were admission to pediatric or neonatal intensive care unit, palliative care, or psychiatry, known eating disorders, rehospitalization, prematurity < 1 month of corrected age, and condition leading to anasarca (severe liver, renal or cardiac failure). Informed consent was obtained from participants and/or their caregivers at admission. To ensure research standards among institutions, one coordinator was appointed at each site and received training on forms, questionnaires, measurements, and anthropometric devices. The medical and surgical ward staff was informed about the research project and particularly about the recruitment aspect. The study was approved by the Ethics Review Boards of the CHU Sainte-Justine (#2016–1267). Informed consent was obtained for all participants and parents/legal guardians.

### Inpatient data collection

Between twenty-four to forty-eight hours of hospital admission, a standardized admission form was completed. Information related to medical history, demographics and anthropometric measurements, i.e., weight, height and mid-upper arm circumference (MUAC), was collected. Moreover, medical history including admission diagnosis, underlying medical condition and condition severity was reported. Underlying medical condition refers to any other conditions that were present or documented in the medical chart but were not the primary reason for admission. Condition severity was categorized as either mild (grade 1), moderate (grade 2), or severe (grade 3) based on clinical stress factors related to disease, diagnosis or planned medical procedure [39, 40]. During hospital stay, food consumption was monitored to calculate the energy intake. Consumption of hospital meals was recorded on a standardized form provided directly on the meal tray. The form was filled by caregivers as instructed by the site coordinator. Caregivers were instructed to record consumption of all food items and beverages on a 6-point scale (none, < 25%, 25%, 50%,75%, and 100%) for 3 consecutive days during the first week of admission, then food intake was measured 2 days per week until discharge. If other food items were consumed, caregivers had to report the information in a food journal. A 24-h food recalls were also used if caregivers were not present during mealtimes. Compilations were then completed by the site coordinator. The daily energy intake was expressed as a percentage of estimated energy requirements (%EER), which was calculated based on sex, age, medical diagnosis, and severity of condition [41–46]. For all patients, the level of activity was considered sedentary during hospital stay. Dietitian visits were documented. Upon discharge, weight was measured and weight variation during hospital stay was computed.

### Nutritional risk and status evaluation

Upon admission, participants were screened for nutritional risk using the STRONGkids form. Nutritional status was assessed using the SGNA tool and anthropometric measurements. Height/length-for-age z-score (HAZ), weight-for-length z-score (WFLZ), BMI-for-age z-score (BMIAZ) were calculated with the growth standard and charts of the World Health Organization (WHO). MUAC z-score (MUACZ) determination was based on U.S. populational data proposed by Abdel-Rahman et al. [47]. Mild, moderate and severe malnutrition was defined as having at least 1 growth parameter with a z-score of < -1 < -2 and < -3 respectively according to the criteria established by the Academy of Nutrition and Dietetics/American Society for Parenteral and Enteral Nutrition [2]. Weight was measured every 2 days throughout hospital stay. Nutritional status according to anthropometrics was determined based on the most severe z-score among the 4 associated variables (HAZ, WFLZ, BMIAZ and MUACZ).

### Post-discharge data collection

Thirty days after hospital discharge, a short questionnaire was conducted by the site coordinator to caregivers over the phone. The questionnaire consisted of multiple-choice questions on the child’s appetite level, eating habits, weight loss and health care professional visits. Binary questions (yes/no) included the occurrence of post-discharge complications and/or hospital readmission. The nature of complications that occurred following discharge (i.e. not present during hospitalisation) was documented. Participants who were readmitted for scheduled elective medical intervention, such as chemotherapy or surgery, were included in the "no complication" category at post-discharge.

### Statistical analyses

SPSS version 26.0 was used for statistical analyses, using frequencies to describe the cohort. The primary outcome studied was post-discharge complications. Hospital readmission was tested as a secondary outcome. Pearson’s chi-squared tests were performed to assess the relationship between both outcomes (post-discharge complications and hospital readmission) and various nutritional factors: nutritional risk, nutritional status according to different evaluation tools and measures, appetite level and energy intake. Logistic regressions were used for estimations of odds ratios (ORs) and corresponding 95% confidence intervals (CIs) to quantify the odds of post-discharge complications and hospital readmission regarding the selected independent variables [patient’s characteristics (sex, age category, underlying medical condition, diagnosis at admission and length of stay), nutritional factors measured at admission (nutritional risk and status evaluation), during hospital stay (energy intake and weight loss > 5%) and at post-discharge (appetite level and weight loss)]. To quantify the strength of the relationship of each independent variable with the two outcomes, univariate logistic regression model was used as crude analysis..The adjusted analysis included covariates selected based on the literature and clinical relevance: sex, age, underlying medical condition and center of care. For all regression analyses (crude and adjusted) of both outcomes, nutritional risk assessed with the STRONGkids tool was grouped into 2 categories: low or high risk. Participants who had a moderate risk score were included in the high-risk group. SGNA-based nutritional status was also grouped into 2 categories: well-nourished or malnourished. The malnourished group included both the moderately and severely malnourished. Frequencies were used to describe the type of post-discharge complication based on the occurrence of readmission. All missing data including dietary intake were considered missing at random and analysis was conducted on the available data collected for each participant (without imputation). For all analyses, a *P*-value < 0.05 was considered statistically significant.

## Results

### Study population and characteristics of participants

In total, 371 participants were initially recruited to participate in the study. Eleven participants were removed from the analyses due to missing data entry. Therefore, the final sample size was 360 patients. Participants’ main characteristics are described in Table 1. Median age was 6.1 years and median length of stay was 5 days. Most participants were hospitalized at CHU Sainte-Justine in Montreal, Quebec (36.4%) and at Alberta Children's Hospital in Calgary, Alberta (36.1%). The primary reason for admission by system was gastrointestinal/hepatic (17.9%), respiratory (17.4%), infectious (13.7%) and hematology/oncology (11.8%).

**Table 1:** Characteristics of participants at admission and during hospital stay

Characteristics	All (*n* = 360)	
Male sex, n (%)	186	(51.7)
Age, y, median (IQR)	6.07	(1.81–11.7)
Age category, n (%)		
< 2 y	113	(31.4)
2–5 y	84	(23.3)
6–12 y	95	(26.4)
13–18 y	68	(18.9)
Center of care, n (%)		
CHU Sainte-Justine	131	(36.4)
Alberta Children Hospital	130	(36.1)
BC Women and Children	6	(1.7)
Kingston General Hospital	23	(6.4)
IWK Health Centre	70	(19.4)
Admission diagnosis, n (%)	357	
Cardiovascular	8	(2.2)
Gastrointestinal/hepatic	64	(17.9)
Genitourinary	14	(3.9)
Respiratory	62	(17.4)
Musculoskeletal	29	(8.1)
Neurological	27	(7.6)
Autoimmune disease	4	(1.1)
Metabolic disorder	6	(1.7)
Trauma (including burns)	6	(1.7)
Infectious	49	(13.7)
Renal	15	(4.2)
Hematology/oncology	42	(11.8)
Developmental	11	(3.1)
Other	20	(5.6)
Underlying medical condition, n (%)		
Yes	195	(54.2)
Energy intake^a^, n (%)	245	
< 50% EER	171	(69.8)
≥ 50% EER	74	(30.2)
Weight loss ≥ 5% during hospital stay, n (%)	287	
Yes	32	(11.1)
Length of stay, d, median (IQR)	5	(3–7)

Demographic (sex, age) and medical (admission diagnosis and underlying medical condition) characteristics were collected using a standardized admission form. Underlying medical condition refers to any other conditions that were present or documented in the medical chart but were not the primary reason for admission. Food consumption was monitored by caregivers during hospital stay and revised by the site coordinator at the following frequency: on 3 consecutive days for the first week of admission and for 2 days per week until discharge

*D* day, *EER* Estimated energy requirements, *IQR* interquartile range, *y* years

^a^Daily dietary intake was expressed as a percentage of energy intake compared to estimated energy requirement, which was calculated according to age, sex, medical diagnosis and severity of condition. For all patients, the level of activity was considered sedentary

At admission (Table 2), most participants (87.0%) were at medium or high nutritional risk using the STRONGkids nutritional risk screening, and 123 (34.7%) were moderately or severely malnourished according to the SGNA. When looking at anthropometric measurements, 56 (16.2%) were classified as moderately or severely malnourished based on having at least one anthropometric measure with a z-score < -2. Lastly, according to the MUACZ, 21.3% of participants had mild malnutrition (z-score: < -1 to -1.99), 6.0% had moderate malnutrition (z-score: < -2 to -2.99), and 5.2% had severe malnutrition (z score < -3).

**Table 2:** Nutritional risk and status assessment of participants at admission

Assessment	Classification, n (%)			
*Nutritional risk*		**Low**	**Medium**	**High**
STRONGkids, *n* = 355		46 (13.0)	222 (62.5)	87 (24.5)
*Nutritional status*	**Well-nourished**		**Malnutrition**	
		**Mild**	**Moderate**	**Severe**
SGNA, *n* = 354	231 (65.3)	N/A	103 (29.1)	20 (5.6)
HAZ^a^, *n* = 307	286 (93.2)	N/A	N/A	21 (6.8)
WFLZ^a^, *n* = 82	65 (79.3)	6 (7.3)	5 (6.1)	6 (7.3)
BMIAZ^a^, *n* = 225	181 (80.4)	26 (11.6)	15 (6.7)	3 (1.3)
MUACZ^a^, *n* = 267	180 (67.5)	57 (21.3)	16 (6.0)	14 (5.2)
Anthropometrics^b^, *n* = 347	225 (64.8)	66 (19.0)	20 (5.8)	36 (10.4)

The nutritional risk and status of participants was determined by various methods, including validated tools (STRONGkids and SGNA) and anthropometric measurements (HAZ, WFLZ, BMIAZ and MUACZ) completed at the time of admission

*BMIAZ* BMI-for-age z-score, *HAZ* height/length-for-age z-score, *MUACZ* mid upper-arm circumference z-score, *N/A* non applicable, *SGNA* subjective global nutrition assessment, *WFLZ* weight-for-length z-score

^a^Mild, moderate and severe classification of malnutrition status was defined as having a z-score of < -1, < -2 and < -3, respectively, according to the criteria established by the Academy of Nutrition and Dietetics/American Society for Parenteral and Enteral Nutrition (AND/ASPEN). ^b^Nutritional status according to anthropometrics was determined based on the most severe z-score among the 4 associated variables (HAZ, WFLZ, BMIAZ and MUACZ)

Following discharge, 23.7% and 30.4% caregivers reported that their children had poor appetite and lost weight, respectively (Table 3). Post-discharge complications occurred in 24.1% of the cohort classified as either acute infection (51%), gastrointestinal symptoms (26%) or worsening of the underlying medical condition (23%) and 19.5% were readmitted to hospital within 30 days.

**Table 3:** Post-discharge characteristics of participants

Characteristics	All (*n* = 344)	
Weight loss, n (%)	273	
Yes	83	(30.4)
Poor appetite level, n (%)	257	
Yes	61	(23.7)
Eating socially, n (%)	264	
Never	13	(4.9)
Sometimes	18	(6.8)
Often	35	(13.3)
Always	153	(58.0)
N/A (breast-fed/formula-fed)	45	(17.0)
Dietitian visit, n (%)	268	
Yes	51	(19.0)
Doctor visit, n (%)	271	
Yes	220	(81.2)
Discussed nutrition with health care professional, n (%)	265	
Yes	43	(16.2)
Complications, n (%)		
Yes	83	(24.1)
Hospital readmission, n (%)		
Yes	67	(19.5)

### Primary outcome: post-discharge complications and associated nutritional factors

**Fig. 1:**
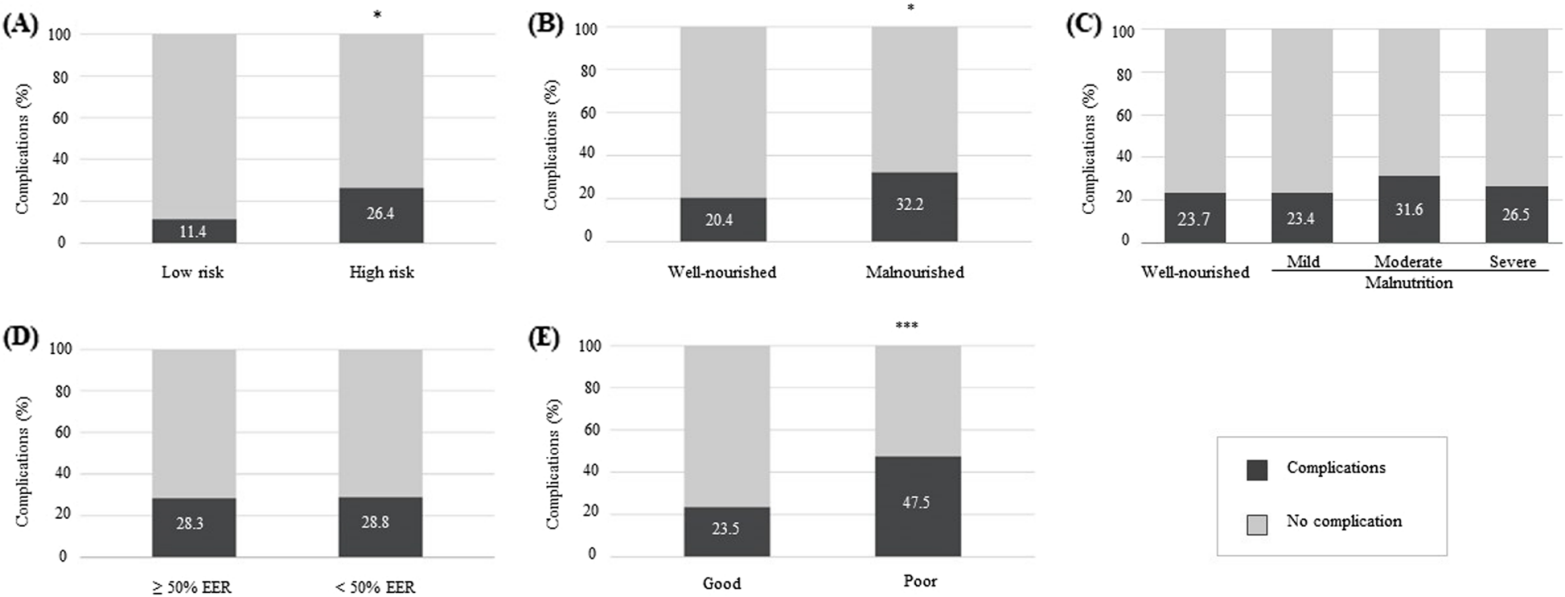
Nutritional factors and post-discharge complications. The proportion of participants experiencing complications post-discharge was defined according to: (**A**) Malnutrition risk score using the STRONGkids tool; (**B**) Nutritional status using the SGNA questionnaire; (**C**) Nutritional status measured with anthropometrics (height/length-for-age z-score, weight-for-length z-score, BMI-for-age z-score, mid upper-arm circumference z-score) where mild, moderate and severe malnutrition was defined as having at least 1 growth parameter with a z-score of < -1, < -2 and < -3 respectively; (**D**) Percentage of energy intake/estimated energy requirement during hospital stay; (**E**) Appetite level after discharge. *P < 0.05; ***P < 0.001 using Chi-squared test

Factors associated with the occurrence of post-discharge complications are presented in Fig. 1 and Table 4. Individuals at high nutritional risk experienced more complications than their lower nutritional risk counterparts (26.4% vs 11.4%; χ^2^ = 4.663; *P* < 0.05) and were nearly 3 times more likely to experience complications post-discharge (OR, 2.85; 95% CI, 1.08–7.54; *P* = 0.035). Nutritional status assessed with the SGNA was also related to post-discharge complications: being malnourished was associated with a higher prevalence of post-discharge complications (32.2% vs 20.4%; χ^2^ = 5.834; *P* < 0.05) and almost doubled the risk of developing complications within 30 days following hospital stay (OR, 1.92; 95% CI, 1.15–3.20; *P* = 0.013). No difference was found in post-discharge complication rates between patients with or without an underlying medical condition (crude analysis) and in those considered well-nourished, mildly, moderately and severely malnourished (23.7% vs 23.4% vs 31.6% vs 26.5%; χ2 = 0.696; *P* = 0.879) when anthropometric criteria were used to determine their nutritional status. As the "mild" malnutrition category is controversial in clinical practice, the analysis was repeated after grouping the patients from this category in the "well-nourished" group. Despite this reclassification, no association between post-discharge complications and nutritional status was identified (data not shown). During hospital stay, participants who had a low food intake (< 50% EER) had a similar complication rate to those who met their energy requirements (28.3% vs 28.8%, χ^2^ = 0.05; *p* = 0.942). However, participants who reported poor appetite post-discharge had more complications than children with a good appetite level (47.5% vs 23.5%; χ^2^ = 12.669; *p* < 0.001). Having a poor appetite tripled the risk of experiencing complications (OR, 2.96; 95% CI, 1.59–5.50; *p* < 0.001).

**Table 4:** Factors associated with post-discharge complications

Factors	OR (95% CI), crude	*P* value	OR (95% CI), adjusted	*P* value
Sex		0.091		
Female	Reference			
Male	0.97 (0.59–1.59)			
Age category		0.69		
Adolescents (13–18 y)	Reference			
Children (6–12 y)	1.09 (0.53–2.23)			
Preschoolers (2–5 y)	1.30 (0.68–2.49)			
Infants (< 2 y)	1.04 (0.53–2.07)			
Underlying medical condition		0.59		
No	Reference			
Yes	0.87 (0.53–1.43)			
Diagnosis at admission		0.53		0.457
Gastrointestinal/hepatic	Reference		Reference	
Respiratory	1.54 (0.77–3.09)		0.58 (0.25–1.36)	
Infectious	0.95 (0.45–2.01)		0.61 (0.24–1.57)	
Hematology/oncology	0.92 (0.40–2.16)		1.22 (0.51–2.89)	
Developmental	1.80 (0.83–3.89)		0.53 (0.10–2.83)	
Others^a^	0.90 (0.18–4.48)		0.64 (0.32–1.30)	
STRONGkids score at admission		0.037		0.035
Low risk	Reference		Reference	
High risk	2.79 (1.06–7.34)		2.85(1.08–7.54)	
SGNA score at admission		0.016		0.013
Well-nourished	Reference		Reference	
Malnourished	1.86 (1.12–3.08)		1.92(1.15–3.20)	
MUACZ^b^ at admission		0.863		0.865
Well-nourished	Reference		Reference	
Mild malnutrition	1.09 (0.53–2.24)		1.11 (0.53–2.29)	
Moderate malnutrition	1.25 (0.38–4.14)		1.27 (0.37–4.38)	
Severe malnutrition	0.57 (0.12–2.67)		0.59 (0.12–2.78)	
Nutritional status^c^ at admission		0.876		0.877
Well-nourished	Reference		Reference	
Mild malnutrition	0.98 (0.51–1.90)		0.99 (0.51–1.91)	
Moderate malnutrition	1.48 (0.54–4.10)		1.47 (0.53–4.11)	
Severe malnutrition	1.16 (0.51–2.64)		1.18 (0.52–2.71)	
Energy intake^d^ during hospital stay		0.94		0.78
≥ 50% EER	Reference		Reference	
< 50% EER	0.98 (0.53–1.80)		1.15 (0.44–3.04)	
Weight loss ≥ 5% during hospital stay		0.692		0.715
No	Reference		Reference	
Yes	1.18 (0.52–2.70)		1.17 (0.51–2.69)	
Length of stay, per day	1.04 (0.99–1.08)	0.098	1.04 (0.99 – 1.08)	0.116
Weight loss post-discharge		0.791		0.502
No	Reference		Reference	
Yes	1.08 (0.61–1.92)		0.81 (0.44–1.49)	
Appetite level post-discharge		0.001		0.001
Good	Reference		Reference	
Poor	2.95 (1.60–5.41)		2.96 (1.59–5.50)	

Model included age category, sex, underlying medical condition and center of care

*EER* Estimated energy requirements, *MUACZ* mid-upper arm circumference z-score, *SGNA* Subjective global nutritional assessment

^a^Other diagnoses included cardiovascular, genitourinary, autoimmune disease, renal, metabolic, and musculoskeletal disorders and trauma

^b^Mid-upper arm circumference was measured at admission. Values were inserted in age and sex specific equations designed for U.S. pediatric population. A z-score < -1 indicated mild malnutrition; < -2, moderate malnutrition; < -3 severe malnutrition according to the criteria established by the Academy of Nutrition and Dietetics/American Society for Parenteral and Enteral Nutrition (AND/ASPEN)

^c^Nutritional status according to anthropometrics was determined based on the most severe z-score among the 4 associated variables (HAZ, WFL, BMIAZ and MUACZ)

^d^Daily dietary intake was expressed as a percentage of energy intake compared to estimated energy requirement, which was calculated according to age, sex, medical diagnosis and severity of condition. For all patients, the level of activity was considered sedentary

### Secondary outcome: readmissions and associated nutritional factors

**Fig. 2:**
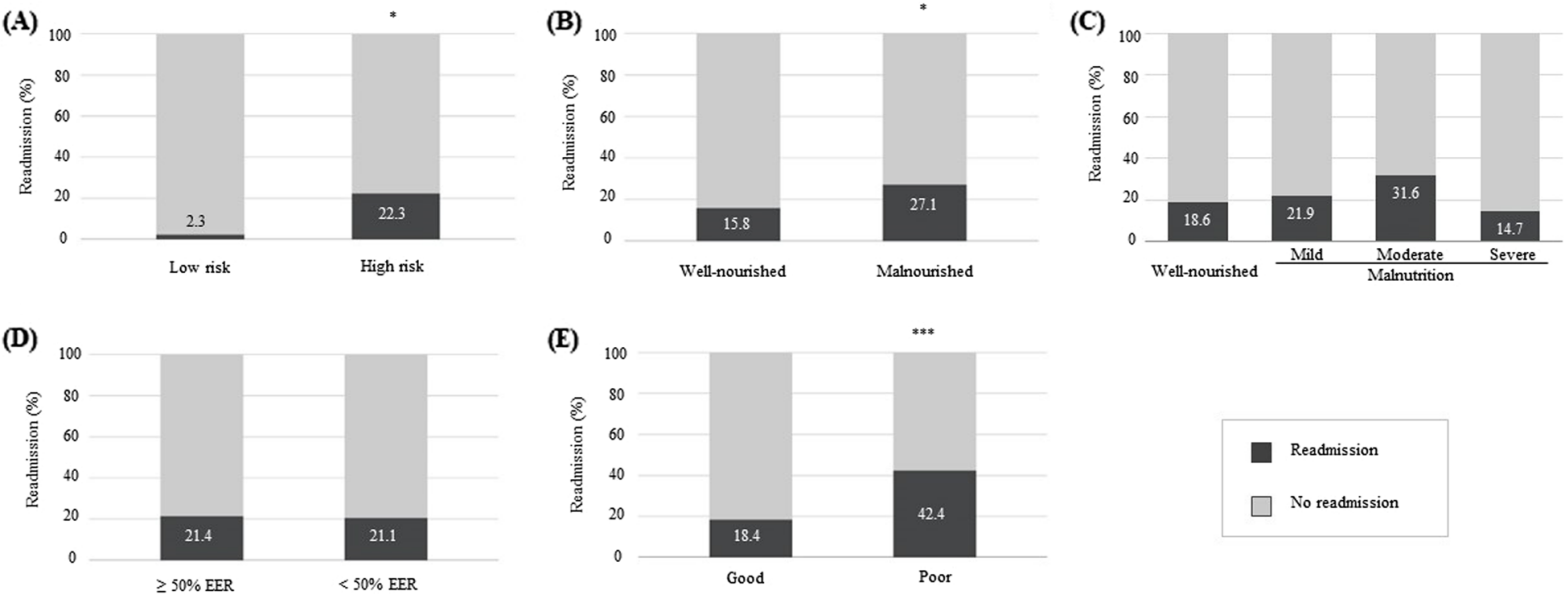
Nutritional factors and hospital readmission. The proportion of participants experiencing complications post-discharge was compared according to: (**A**) Malnutrition risk score using the STRONGkids tool; (**B**) Nutritional status using the SGNA questionnaire; (**C**) Nutritional status measured with anthropometrics (weight-for-height z-score, height-for-age z-score, BMI-for-age z-score, mid upper-arm circumference z-score) where mild, moderate and severe malnutrition was defined as having at least 1 growth parameter with a z-score of < -1, < -2 and < -3 respectively; (**D**) Percentage of energy intake/energy requirement during hospital stay; (**E**) Appetite level after discharge. *P < 0.05; ***P < 0.001 using Chi-squared test

Factors associated with hospital readmission are detailed in Fig. 2 and Table 5. The proportion of children readmitted following discharge was higher in the high nutritional risk compared to low risk (22.3% vs 2.3%; χ^2^ = 9.708; *p* < 0.05) and patients with high nutritional risk were 10 times more likely to be readmitted than those identified with a low risk. SGNA nutritional status was associated with readmissions, as the percentage was higher in participants with poor status compared to those with normal status (27.1% vs 15.8%, χ^2^ = 6.174; *p* < 0.05) and patients with a malnourished status with the SGNA were 1.95-time more likely to be readmitted compared to the well-nourished (95% CI, 1.12–3.39, *p* = 0.017). Underlying medical conditions (crude analysis), nutritional status measured with anthropometrics and energy intake were not associated with readmissions in both crude and adjusted analyses. Similar to post-discharge complications, no relationship was revealed with readmission despite the reclassification of participants with “mild” malnutrition into the “well-nourished” group (data not shown). Children with poor appetite after discharge experienced more readmissions (42.4% vs 18.4%, χ^2^ = 14.360; *p* < 0.001) and were 3.68-time more likely to be readmitted compared to those who had a good appetite following discharge.

**Table 5:** Factors associated with hospital readmission

Factors	OR (95% CI), crude	*P* value	OR (95% CI), adjusted	*P* value
Sex		0.939		
Female	Reference			
Male	0.98 (0.57–1.67)			
Age category		0.224		
Adolescents (13–18 y)	Reference			
Children (6–12 y)	0.42 (0.18–0.97)			
Preschoolers (2–5 y)	0.71 (0.33–1.55)			
Infants (< 2 y)	0.81 (0.39–1.65)			
Underlying medical condition^±^		0.329		
No	Reference			
Yes	1.31 (0.76–2.25)			
Diagnosis at admission		< 0.001		< 0.001
Gastrointestinal/hepatic	Reference		Reference	
Respiratory	0.46 (0.17–1.25)		0.50 (0.18–1.41)	
Infectious	0.57 (0.20–1.64)		0.62 (0.21–1.86)	
Hematology/oncology	5.32 (2.23–12.7)		5.63 (2.27–13.95)	
Developmental	0.40 (0.05–3.47)		0.46 (0.051–4.04)	
Others^a^	0.46 (0.20–1.05)		0.46 (0.20–1.06)	
STRONGkids score at admission		0.014		0.012
Low risk	Reference		Reference	
High risk	12.34 (1.67–91.30)		13.23 (1.77–98.64)	
SGNA score at admission		0.14		0.017
Well-nourished	Reference		Reference	
Malnourished	1.98 (1.15–3.40)		1.95 (1.12–3.39)	
MUACZ^b^ at admission		0.588		0.576
Well-nourished	Reference		Reference	
Mild malnutrition	1.17 (0.57–2.40)		1.15 (0.55–2.38)	
Moderate malnutrition	1.34 (0.40–4.44)		1.14 (0.34–3.90)	
Severe malnutrition	0.283 (0.04–2.23)		0.25 (0.03–2.01)	
Nutritional status^c^ at admission		0.471		0.480
Well-nourished	Reference		Reference	
Mild malnutrition	1.23 (0.62–2.43)		1.22 (0.61–2.43)	
Moderate malnutrition	2.02 (0.72–5.64)		1.97 (0.70–5.55)	
Severe malnutrition	0.75 (0.28–2.07)		0.73 (0.26–2.01)	
Energy intake^d^ during hospital stay		0.89		0.282
≥ 50% EER	Reference		Reference	
< 50% EER	0.95 (0.48–1.88)		0.56 (0.19–1.61)	
Weight loss ≥ 5% during hospital stay		0.488		0.439
No	Reference		Reference	
Yes	1.36 (0.57–3.21)		1.42 (0.59–3.41)	
Length of stay, per day	1.04 (1.00–1.09)	0.063	1.04 (1.00–1.09)	0.058
Weight loss post-discharge		0.79		0.745
No	Reference		Reference	
Yes	0.92 (0.50–1.71)		1.12 (0.58–2.15)	
Appetite loss post-discharge		< 0.001		< 0.001
No	Reference		Reference	
Yes	3.26 (1.74–6.14)		3.68 (1.90–7.14)	

Model included age category, sex, underlying medical condition and center of care

*EER* Estimated energy requirements, *MUACZ* mid-upper arm circumference z-score, *SGNA* Subjective global nutritional assessment

^a^Other diagnoses included cardiovascular, genitourinary, autoimmune disease, renal, metabolic, and musculoskeletal disorders and trauma

^b^Mid-upper arm circumference was measured at admission. Values were inserted in age and sex specific equations designed for U.S. pediatric population. A z-score < -1 indicated mild malnutrition; < -2, moderate malnutrition; < -3 severe malnutrition according to the criteria established by the Academy of Nutrition and Dietetics/American Society for Parenteral and Enteral Nutrition (AND/ASPEN)

^c^Nutritional status according to anthropometrics was determined based on the most severe z-score among the 4 associated variables (HAZ, WFLZ, BMIAZ and MUACZ)

^d^Daily dietary intake was expressed as a percentage of energy intake compared to estimated energy requirement, which was calculated according to age, sex, medical diagnosis and severity of condition. For all patients, the level of activity was considered sedentary

**Fig. 3:**
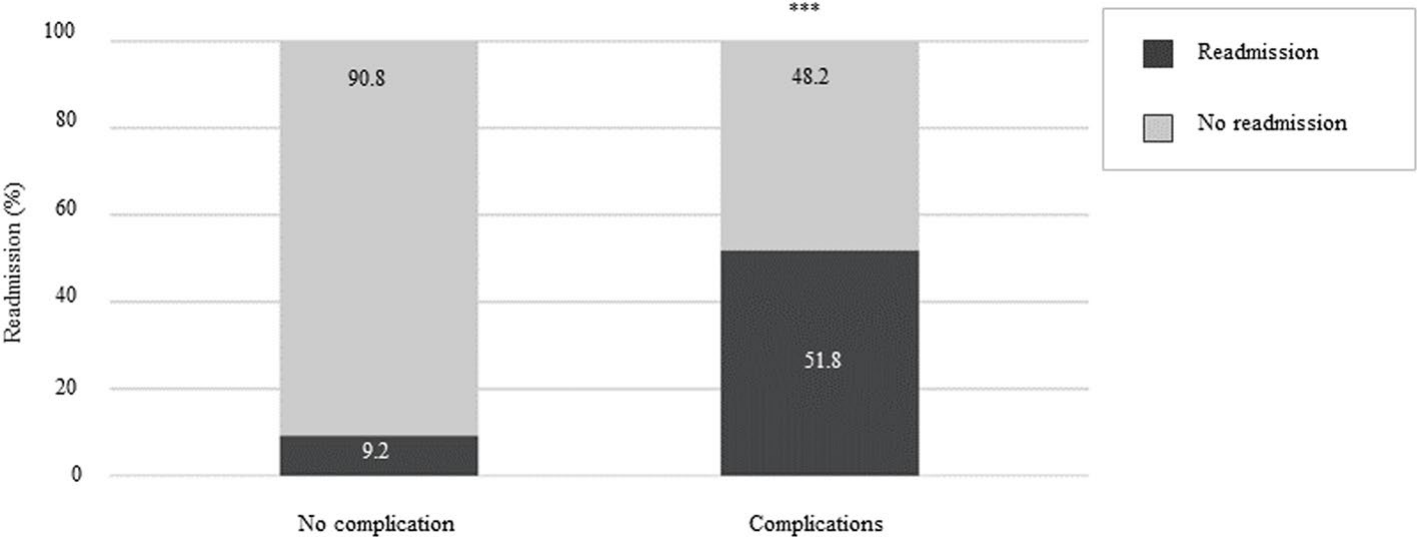
Post-discharge complications and hospital readmission. Data were computed according to the presence (*n* = 83) or absence (*n* = 261) of post-discharge complications. ***P < 0.001 using Chi-squared test

**Fig. 4:**
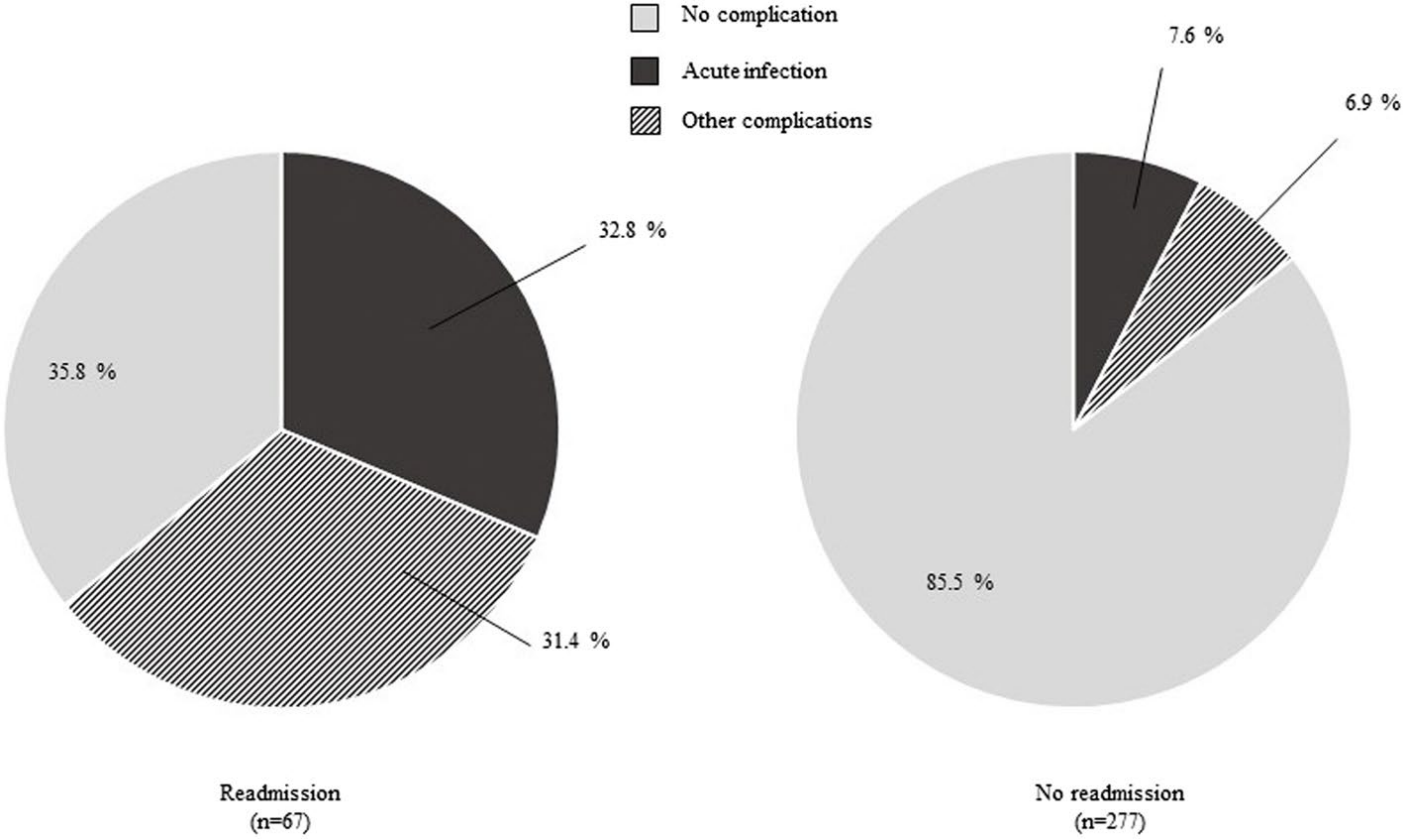
Hospital readmission according to the type of post-discharge complication. Participants were grouped according to the occurrence of hospital readmission (*n* = 67) or no readmission (*n* = 277). Each group was subdivided per complication type: acute infection, other complications, no complications. Other complications included mainly gastrointestinal symptoms and worsening of known medical condition

Participants who had complications post-discharge were more likely to be readmitted to the hospital (51.8% vs 9.2%, χ^2^ = 72.909; *p* < 0.001) (Fig. 3). Figure 4 illustrates the type of complications according to readmission status. In the readmitted group, 64.2% of children experienced complications. Reasons documented for readmission were acute infections (32.8%) and other complications (31.4%), which included gastrointestinal symptoms and worsening of current medical condition. In the no readmission group, most participants did not experience complications (85.5%), and those with complications were treated in community settings.

## Discussion

To our knowledge, this is the first study that has examined post-discharge consequences in relation to children’s nutritional risk and malnutrition status in a high resource country. Our results revealed that a high nutritional risk score and a poor nutritional status obtained respectively with the STRONGkids and the SGNA questionnaires were associated with post-discharge complications and hospital readmission. In contrast, assessment of nutritional status using anthropometric parameters revealed no association with post-discharge complications or hospital readmission. A poor appetite following hospital stay was strongly associated with the development of complications. Complications reported included acute infections, gastrointestinal symptoms and worsening of known medical condition.

Post-discharge complications, hospital readmission and mortality in relation to malnutrition have been studied in pediatric populations from low to high resource settings [10, 26, 48–52]. In low resource settings, it has been proposed that malnutrition impacts immunity by modulating the intestinal microbiome, reducing gut barrier function, altering regulation of inflammatory cytokines, and decreasing uptake of nutrients, while infection promotes malnutrition by increasing nutrient losses, reducing nutrient uptake and increasing energy expenditure [53]. Nonetheless, in line with previous observations in low resources countries, we found that malnourished children according to SGNA assessment presented a higher rate of post-discharge complications and hospital readmission [26, 50, 54]. Our findings support that a moderately or highly malnourished state identified by the SGNA tool may contribute to host vulnerability. Thus, the higher proportion of post-discharge complications identified in the malnourished participants were predominantly acute infections acquired in the community. A systematic review published by Rytter et al*.* [55] stated that different types of malnutrition are associated with different immunological alterations, however underlying mechanisms are still inadequately understood, showing the need for prospective studies based on current understanding of immunology. Since etiology should be considered in the definition of pediatric malnutrition [1, 2], it would be interesting to study this malnutrition-immunity interrelation in tertiary acute care pediatric populations based on admission diagnosis. As an example, children with diarrhea may be more at risk of micronutrient deficiencies, which can impact their immune status and potentially increase the risk of complications. This could allow for etiology-based interventions, targeting specific malnutrition-promoting pathways.

The STRONGkids tool has been criticized for its low specificity in classifying children according to their nutritional status [13, 56]. In our study, 25.4% of participants were at high risk of malnutrition according to the STRONGkids score. In comparison, a European study conducted in 14 tertiary centers of care, the range of high risk of malnutrition evaluated with the same tool ranged from 5 to 30% [57]. Also, we report an association between nutritional risk screening tool STRONGkids and post-discharge outcomes (complications and readmission). To our knowledge, similar associations have only been described in pediatric populations in Asia (Thailand and China) and Brazil therefore in different socio-economic contexts [58–60]. This is an interesting result as this tool was primarily developed to identify, at admission, patients most at risk of nutrition-related complications during hospitalization and not necessarily post-discharge outcomes [16]. In Canada, as part of the Pediatric Integrated Pathway for Acute Care (P-INPAC), a group of research clinicians recently suggested the use of the STRONGkids tool, the Paediatric Nutrition Screening Tool (PNST) to screen for nutritional risk and anthropometric measurements upon all hospital admission (https://nutritioncareincanada.ca/resources-and-tools/pediatrics). This evidence-based algorithm provides insight on how to prevent, detect and treat malnutrition in acute care settings. Heterogeneity exists amongst Canadians centers and services as many tools are available to screen and assess malnutrition. Having a standardized approach towards pediatric malnutrition could help reduce variability of data nationally, which could guide more specific interventions.

We found that malnutrition status based solely on anthropometric measures at admission was not a factor associated with the outcomes (post-discharge complications and readmission). Accordingly, Secker and Jeejeebhoy highlighted a discrepancy in the association between malnutrition status and complications following surgical intervention in pediatric patients depending on the method used to assess nutritional status. A higher rate of infectious complications was identified in malnourished children when the SGNA was used, while there was no difference when malnutrition was defined based on anthropometric parameters [22]. MUAC is an anthropometric measure of particular interest when weight is unreliable and has been proven to show improvement more readily than BMIAZ [61]. We did not find any association between MUACZ and post-discharge outcomes. However, many studies have demonstrated that malnutrition status at admission based on anthropometric measures is associated with higher in-hospital complications in children such as prolonged hospital stay and increased complication rates [6, 10, 17, 29, 62–65]. The SGNA and STRONGkids screening tools include multiple key domains of malnutrition assessment such as poor growth or stagnant growth and etiologic causes of malnutrition, which is a more comprehensive assessment than anthropometric measurements alone [1, 2, 9, 16, 22]. This may account for the lack of association between complications and anthropometric measurements. Using the WHO criteria alone may not be adequate to identify clinically relevant changes in nutritional status in those with chronic disease [54]. In a high resource setting, this may be particularly relevant, as in the present study a significant proportion of the population had chronic malnutrition in the form of stunting. Also, comparison of 30-day readmission rate and its associated factors with other countries regarding should be made with caution. As such, one of the contributors to all-causes unplanned hospital readmission often cited is whether or not children have access to health insurance [66], a criterion that is less relevant in countries with universal healthcare such as Canada. Finally, in our study, having an underlying medical condition was not associated with post-discharge complications and 30-day readmission. Other studies evaluating post-discharge complications associated with nutritional status in pediatric hospital settings are needed to confirm these findings.

### Nutrition and hospital discharge practices

A study recently published by Huysentruyt et al. examined nutritional screening, assessment, and discharge practices amongst 15 tertiary pediatric care centers in Canada [31]. The main findings suggested that routine nutritional risk screening at admission is not widely adopted. More specifically, only 15% of the participants indicated that nutritional screening was always performed and a high variety in the definition of nutritional screening was reported. Furthermore, standardized protocols for the clinical assessment and management of malnutrition were described as uncommon, with a generalized lack of structure for post-discharge nutritional referrals across the country [31]. With the median length of stay being 3 to 5 days, it is highly suspected that nutritional status optimization is not completely achieved during hospital stay [31]. This is in line with our findings, showing that nutritional risk and status during hospitalization impact post-discharge complications and readmissions. These results suggest that nutritional discharge practices should be enhanced to continue nutritional status optimization post-discharge. Basic nutrition care, such as nutritional risk screening, should be incorporated in a multidisciplinary approach in the community. Also, a simple question on appetite level (good vs. poor) seems to be a good indicator of clinical evolution after hospital discharge. Nutritional discharge practices could be examined, as well as non-dietitian health care professionals’ nutrition knowledge and practices. Investigating resources in the transition from hospital to home and how standard nutrition services and interventions in the community benefit malnourished pediatric patients should be considered in future research.

### Strengths and limitations

The strengths of this study include its multicentered approach, design, and execution. Different nutritional screening and assessment methods were included, which allowed for a detailed portrait of malnutrition in this population. Limitations include a possible selection bias as each center could not accurately document the total number of patients screened and the specific reasons of ineligibility for study exclusion. Post-discharge parental reported questionnaires were subjective in nature, as was the feedback from caregivers. Additionally, when looking at factors associated with hospital readmission, patients with a diagnosis in hematology/oncology experience more readmissions than patients with other admission diagnoses. This is most likely attributed to chemotherapy treatments causing febrile neutropenia and requiring hospital readmission. Next, when computing logistic regressions, the small number of participants included in some categories led to large confidence intervals. The original tools were not validated with these categories. Lastly, these variables were measured at hospital admission; in future work, it would be relevant to have objective measure of these nutritional factors at discharge or post-discharge to allow for a deeper understanding of nutritional status evolution.

## Conclusion

This study confirms the association between malnutrition risk and status and post-discharge complications in pediatric Canadian patients. It also reveals that inpatient children identified with high malnutrition risk and malnourished according to SGNA have worst post-discharge outcomes, notably more acute infections, and short-term hospital readmissions. This was not identified with single anthropometric measurements highlighting the value of a more comprehensive nutritional assessment. Enhancing nutritional care and monitoring during admission, and at discharge to the community may be an area of outcome optimization.

### Supplementary Information

Supplementary Material 1.

## Data Availability

The datasets generated during and/or analysed during the current study are available from the corresponding author on reasonable request.

## Abbreviations

BMIAZ: BMI-for-Age Z-score
CHU: Centre Hospitalier Universitaire
CI: Confidence Intervals
EER: Estimated Energy Requirements
HAZ: Height/length-for-Age Z-score
ICD-10-CA: International Statistical Classification of Diseases and Related Health Problems, 10th Revision, Canada
LOS: Length Of Stay
MUAC: Mid-Upper Arm Circumference
MUACZ: MUAC Z-score
OR: Odds Ratio
SGNA: Subjective Global Nutrition Assessment
STRONGkids: Screening Tool for Risk Of impaired Nutritional status and Growth
WFLZ: Weight-For-Length Z-score
WHO: World Health Organization
