# Supplementary material for: Post-discharge complications and hospital readmissions are associated with nutritional risk and malnutrition status in a cohort of Canadian pediatric patients
Source: BMC Pediatr. 2024 Jul 23;24:469. doi: 10.1186/s12887-024-04941-6 (PMC11265476; doi:10.1186/s12887-024-04941-6)
Supplement: Supplementary file 1 — Supplementary Material 1. [file 12887_2024_4941_MOESM1_ESM.pdf]

Patient Code _______________
Date _ _ / _ _ / _ _ _ _

Canadian Malnutrition Task Force | le Groupe de travail canadien sur la malnutrition

Patient Admission Data Tracking Form for the Paediatric Study

Admission Information	Result
**Hospital Ward(s)**<br>{note movement among wards and dates if this occurs}	
**Date of Admission**	

Patient Demographics	Coding Directions	Result
**Gender**	M = 1<br>F = 0	
**Date of Birth**<br>{add age as well}		
**Ethnicity**<br>*"What ethnic group do you identify with?"*<br>{code based on response, more than one code can be used, e.g. African Canadian would be 6,1 – use a comma to separate}<br><br>{provide details if "other" is used}	Canadian = 1<br>European = 2<br>West Asian = 3 (Iranian, Afghan)<br>South Asian = 4 (Pakistani, Indian)<br>East and Southeast Asian = 5 (Vietnamese, Chinese, Korean, Japanese)<br>African = 6<br>Pacific Islands = 7<br>Central / South American = 8<br>Caribbean = 9<br>Aboriginal / Native = 10<br>Arab = 11<br>Other = 12	
**Living Arrangements**<br>{provide details if "other" is used }	Lives with both parents/guardians = 7<br>Lives with single parent/guardian = 8<br>Lives alone = 1<br>Residential care (eg. foster care, boarding school, orphanage care, child disability care)= 9	

Revised October 19, 2012

Patient Code _______________
Date _ _/ _ _/ _ _ _ _

Patient Demographics	Coding Directions	Result
	Nursing home (nursing care required for ADL) = 5<br>Other = 6	
**Food related activities of daily living**<br>*Who is responsible for grocery shopping?*<br>*Who is responsible for cooking?*<br>{two questions requires two sets of codes, record all responses indicated, separate responses with a comma, e.g. Parents does the shopping code as 7; Patient and parents share the cooking code as 1,7}<br><br>{provide details if "other" is used}	Parents or guardians = 7<br>Patient = 1<br>Other family = 4<br>Friend = 8<br>Community support services = 6 (i.e. Meals on Wheels, congregate dining, grocery service, homemaking support)<br>Other = 9	**Shopping**<br><br>**Cooking**
**{Questions 1 to 5 applies to infants and children who eat solid food.}**<br><br>1) How many meals and snacks does the patient usually eat each day?<br><br>{Provide details if "other" is used}	0 = 1<br>1 = 2<br>2 = 3<br>3 = 4<br>Other = 5	**Meals:**<br><br>**Snacks:**
2) Does the patient drink cow's milk? (if no, skip to question 5)	No = 0<br>Yes = 1	
3) Please specify the type of milk consumed.	Whole milk = 1<br>2% milk = 2<br>1% milk or skim milk = 3	
4) How many servings of milk (serving size = 1 cup) does the patient usually consume?	3 or more servings per day = 1<br>2 servings per day = 2<br>1 serving per day = 3<br>2-6 servings per week = 4<br>1 serving per week or less = 5<br>Rarely/never (specify reason) = 6	

Patient Code _______________
Date _ _/_ _/_ _ _ _

Patient Demographics	Coding Directions	Result
5) How many servings of yogurt (serving size = 3/4 cup) or cheese (serving size = 50 g) does the patient usually consume?	3 or more servings per day = 1<br>2 servings per day = 2<br>1 serving per day = 3<br>2-6 servings per week = 4<br>1 serving per week or less = 5<br>Rarely/never (specify reason) = 6	**Yogurt:**<br><br><br>**Cheese:**
**Have you previously consulted a dietitian?**<br><br>If yes, for what reason?	No = 0<br>Yes = 1<br><br>List what the patient/parent reports	
**Do you follow a special diet at home?**<br><br>If yes, what is the diet?	No = 0<br>Yes = 1<br><br>List what the patient/parent reports	
**Oral Nutrition Supplement Use Prior to Hospitalization**<br>{*Were you taking any nutrition supplements or meal replacements before your hospitalization?* If needed, provide names of examples, such as Ensure, Boost, etc. Indicate in writing what they are taking and approximately how much each day}	No = 0<br>Yes = 1	
**Vitamin & Mineral Supplement Use Prior to Hospitalization**<br><br>{Note type & specify reason}	No = 0<br>Yes = 1<br><br>List what the patient/parent reports	
**The highest level of education of either the mother or the father or guardian**	Grade school = 1<br>High school = 2<br>University or college = 3	
**Work of parents or guardians**	One parent/guardian = 1<br>Both parents/guardians = 2<br>Out of work = 3	

Revised October 19, 2012

Patient Code _______________
Date _ _ / _ _ / _ _ _ _

Medical Information	Coding Directions	Result
**Admitting Diagnosis**<br>**Enter admitting diagnosis as written in the medical chart, in addition to the codes below**<br>{if there is more than one primary diagnosis for admission, more than one code can be used, e.g. lung infection and arrhythmia would be 4,1 – use a comma to separate. Note-provide detail if you use "other"}	Cardiovascular = 1<br>Gastrointestinal/hepatic = 2<br>Genitourinary = 3<br>Respiratory = 4<br>Musculoskeletal = 5<br>Neurological = 6<br>Autoimmune disease = 7<br>Metabolic disorder = 8<br>Trauma (including burns) = 10<br>Infectious = 12<br>Renal = 14<br>Hematology/Oncology = 15<br>Developmental = 16<br>Other = 13	
**Other Conditions that are present but not the primary reason for admission**<br>**Write diagnoses provided on the chart in addition to the codes**<br>{Indicate with corresponding number, all that apply, use a comma to separate numbers}	Cardiovascular = 1<br>Gastrointestinal/hepatic = 2<br>Genitourinary = 3<br>Respiratory = 4<br>Musculoskeletal = 5<br>Neurological = 6<br>Autoimmune disease = 7<br>Metabolic disorder = 8<br>Trauma (including burns) = 10<br>Infectious = 12<br>Renal = 14<br>Hematology/Oncology = 15<br>Developmental = 16<br>Other = 13	
**Severity of the patient's condition**<br>{categorized as either mild (grade 1), moderate (grade 2), or severe (grade 3) as defined in protocol}	Mild = 1<br>Moderate = 2<br>Severe = 3	
**Indicate the former health status of the patient**<br>{if history of abnormal growth please provide details}	Previously healthy & following normal growth patterns = 1<br><br>History of abnormal growth patterns = 2	

Revised October 19, 2012

Patient Code _______________
Date _ _ / _ _ / _ _ _ _

Medical Information	Coding Directions	Result
**Medications**<br>{The TOTAL number should equal the TOTAL number of medication as Day 1 (Admission Date) on the Progress Data Tracking Form. Note prn medications, vitamin mineral supplements and antibiotics separately. The first number is the total of all prescribed meds (including prn and supplements). E.g. if the patient is prescribed 7 medications and 3 of these are prn prescriptions the coding should be Total =7, PRN=3.; Note two drugs in combination are counted as 1 preparation}	Number of medications /preparations/ vitamin and mineral supplements patient is currently receiving	**Total=**<br><br>**PRN=**<br><br>**Vitamin/Mineral supplements=**<br><br>**Specify type:**<br><br>**Antibiotics=**
**Past Surgeries**<br>{note types}	Number of surgeries patient has had in past five years	
**Past Acute Care Admissions**<br>{based on patient report}	Number of admissions in past five years	
**Current Cancer**<br>{note type, as well}	Cancer is present = 1<br>Absent = 0	**Type:**
**Past Cancer**<br>{Note type of cancer (s)? How many years ago? Based on patient report}	e.g. 5yrs= lung, 2 years= colon	

Patient Code _______________
Date _ _/_ _/_ _ _ _

Patient Anthropometrics	Coding Directions	Result
**Admission Weight**<br>{measured by other health care professional upon admission to the hospital – if not done leave blank}	kg	
**Current body weight**<br>{patient/family self-report}	kg	
**Usual body weight**<br>{patient/family self-report}	kg	
**Admission Weight**<br>{measured by Site Coordinator using electronic scale or baby scale-note if unable to weigh}	kg	**#1= #2=**<br>**Average=**
**Estimation of weight change**<br>{using the current weight taken by the Site Coordinator and the patient's self-reported weight calculate % weight loss e.g. [current weight – usual body weight]/ usual body weight X 100}		
**Height**<br>{Supine length < 2 years of age<br>Standing height ≥ 2 years of age}<br>*Use arm span if necessary*	cm	**#1= #2=**<br>**Average=**
**Arm span**<br>{for children who are unable to stand or for children with spinal curvature, contractures, or other condition that prevents proper positioning}	cm	**#1= #2=**<br>**Average=**
**Height**<br>{patient/family self-report}	cm	
**Head circumference**<br>{From birth to 2 years of age}	cm	**HC#1= HC#2=**<br>**Average=**
**Mid upper arm circumference**	cm	**MUAC#1= MUAC#2=**<br>**Average=**
**Triceps skinfold thickness**	cm	**TSF#1= TSF#2=**<br>**Average=**
**Edema or ascites**	No = 0<br>Yes = 1	
**STRONGkids**<br>(low, medium or high risk of malnutrition)	After completing the form enter the results in right hand column	
**SGNA**<br>(normal/well nourished, moderately or severely malnourished)	After completing the form enter the results in right hand column	

Revised October 19, 2012

Patient Code _______________

Canadian Malnutrition Task Force | le Groupe de travail canadien sur la malnutrition

Patient Progress and Data Tracking Form for the Paediatric Study

This form is to track all medical and dietary changes during hospitalization. Thus, even if enrollment to study occurs on the second or third day of admission, diet and medication changes that occurred upon and after admission are to be recorded here retrospectively. After enrollment, the chart is reviewed every two days and the weight is re-measured every 2nd day during the first two weeks and weekly thereafter.

Week 1

**Measures**	**Admission Date** {dd/mm/yy}	**Date**	**Date**	**Date**	**Date**	**Date**	**Date**
Weight {kg} {every 2nd day during the first 2 weeks and weekly thereafter}							
Edema or ascites {tick for yes}							
Height {cm} {monthly from the 1st measurement}							
Head circumference {cm} {monthly from the 1st measurement}							
Mid upper arm circumference {weekly from the 1st measurement}							
Triceps skinfold thickness {weekly from the 1st measurement}							

Patient Code _______________

Measures	Admission Date {dd/mm/yy}	Date	Date	Date	Date	Date	Date
Medications {number of meds, including antibiotics, meds added to TPN, and vitamin/mineral supplements– attach daily pharmacy and TPN sheets, if possible}							
Therapeutic antibiotics {number per day}							
Complications (tick the date that patient had complications}*							
New diagnosis {indicate what the diagnosis is}							
New cancer - post admission (tick date of diagnosis)							
ICU (tick each day that the patient is in the ICU)							
Surgery (tick the date that patient had surgery)**							
Diet Order {indicate the diet order each time it changes}							
Did a dietitian see the patient today {as indicated by chart note} Yes = tick, No= leave blank							
Did a diet technician see the patient today {as indicated by chart note} Yes = tick, No= leave blank							
Did a dietetic intern see the patient today {as indicated by chart note} Yes = tick, No= leave blank							
Food Diary {To be filled by parents as protocol} Yes= tick, no= leave blank							
Diet Order for bottle-fed or breast-fed children {tick if either form of feeding is provided and complete the Assessment of Dietary Intake for bottle-fed or breast-fed children}							
Diet Order for EN or TPN {tick if either form of feeding is provided and complete the Patient Progress & Data Tracking Form for En & TPN}							

* Were there any complications that occurred during the patient's hospitalization? Please specify and note if infectious (e.g. upper respiratory infection, urinary tract infection, sepsis, infection of surgical wounds) or non-infectious (e.g. non-infectious surgical complications, dysphagia, constipation, diarrhea, vomiting, convulsion etc.)

____
____
____

** indicate the type of surgery

____
____
____

Patient Code _______________

Week _______

**Measures**	**Admission Date** {dd/mm/yy}	**Date**	**Date**	**Date**	**Date**	**Date**	**Date**
Weight {kg}<br>{every 2nd day during the first 2 weeks and weekly thereafter}							
Edema or ascites<br>{tick for yes}							
Height {cm}<br>{monthly from the 1st measurement}							
Head circumference {cm}<br>{monthly from the 1st measurement}							
Mid upper arm circumference<br>{weekly from the 1st measurement}							
Triceps skinfold thickness<br>{weekly from the 1st measurement}							
Medications<br>{number of meds, including antibiotics, meds added to TPN, and vitamin/mineral supplements– attach daily pharmacy and TPN sheets, if possible}							
Therapeutic Antibiotics<br>{number per day}							
Complications<br>(tick the date that patient had complications}*							
New diagnosis<br>{indicate what the new diagnosis is}							
New cancer - post admission<br>(tick date of diagnosis)							
ICU<br>(tick each day that the patient is in the ICU)							
Surgery<br>(tick the date that patient had surgery)**							
Diet Order<br>{indicate the diet order each time it changes}							

Patient Code _______________

**Measures**	**Admission Date** {dd/mm/yy}	**Date**	**Date**	**Date**	**Date**	**Date**	**Date**
Did a dietitian see the patient today<br>{as indicated by chart note}<br>Yes = tick, No= leave blank							
Did a diet technician see the patient today<br>{as indicated by chart note}<br>Yes = tick, No= leave blank							
Did a dietetic intern see the patient today<br>{as indicated by chart note}<br>Yes = tick, No= leave blank							
Food Diary<br>{To be filled by parents as protocol}<br>Yes= tick, no= leave blank							
Diet Order for bottle-fed or breast-fed children<br>{tick if either form of feeding is provided and complete the Assessment of Dietary Intake for bottle-fed or breast-fed children}							
Diet Order for EN or TPN<br>{tick if either form of feeding is provided and complete the Patient Progress & Data Tracking Form for En & TPN}							

* Were there any complications that occurred during the patient's hospitalization? Please specify and note if infectious (e.g. upper respiratory infection, urinary tract infection, sepsis, infection of surgical wounds) or non-infectious (e.g. non-infectious surgical complications, dysphagia, constipation, diarrhea, vomiting, convulsion etc.)

____

____

____

** indicate the type of surgery

____

____

____

Patient Code _______________
Date _ _ / _ _ / _ _ _ _

Canadian Malnutrition Task Force | le Groupe de travail canadien sur la malnutrition

Patient Discharge & Data Tracking Form for the Paediatric Study

**Patient Anthropometrics**	**Coding Directions**	**Result**
**Discharge Weight**<br>{measured by Site Coordinator using electronic scale or baby scale-note if unable to weigh}	kg	**#1=** **#2=**<br><br>**Average=**
**Height**<br>{if ≥ **1 month from 1st measurement**}<br>{Supine length < 2 years of age<br>Standing height ≥ 2 years of age}<br>*Use arm span if necessary*	cm	**#1=** **#2=**<br><br>**Average=**
**Mid upper arm circumference**<br>{if ≥ **7 days from 1st measurement**}	cm	**MUAC#1=** **MUAC#2=**<br><br>**Average=**
**Triceps skinfold thickness**<br>{if ≥ **7 days from 1st measurement**}	cm	**TSF#1=** **TSF#2=**<br><br>**Average**
**Head circumference**<br>{if ≥ **1 month from 1st measurement**}	cm	**HC#1=** **HC#2=**<br><br>**Average=**
**Edema or ascites**	No = 0<br>Yes = 1	

Revised March 27, 2013

Patient Code _______________
Date _ _ / _ _ / _ _ _ _

Discharge Information	Coding Directions	Result
**Discharged to:**<br>{if you use "other" please provide description}	Home = 1<br>Someone else's home = 2<br>Residential care (eg. foster care, boarding school, orphanage care, child disability care)= 9<br>Nursing home (nursing care required for ADL) = 5<br>Other = 6	
**Patient AND alternate telephone number post discharge, note relationship (e.g. child)**		
**Length of stay**	Calculate number of days in hospital	
**30 day complications**	Yes = 1<br>No = 0<br><br>Specify complications/ note if infectious or non-infectious	
**30 day readmission**	Yes = 1<br>No = 0<br>Provide reason if available	
**Hospital mortality**	Yes = 1<br>No = 0	
**30 day mortality**	Yes = 1<br>No = 0	

Thirty Day Post Discharge Nutrition Care Process Data Collection

The Site Coordinator will phone each patient 30 days after discharge and will ask the following questions, after asking a general "opening" question such as "How are you feeling?"

Who is filling the questionnaire? (Preferably the patient's family or guardian.)

☐ Patient
☐ Family/Guardian/Friend

1. Has your child's weight changed since he/she was discharged?
   - ☐ Yes, and the weight change is significant and/or worries me
     - ☐ Significant weight loss
     - ☐ Significant weight gain
   - ☐ No, his/her weight has stayed within a few pounds
   - ☐ I don't know how much my child weigh or if his/her weight has changed

Patient Code ______________
Date _ _ / _ _ / _ _ _ _

2. How would you describe your child's appetite?
   - □ Very good
   - □ Good
   - □ Fair
   - □ Poor

3. Is your child eating or avoiding certain foods or following a special diet? (give examples e.g. low fat, diabetic, dairy-free diet) If yes, please specify the diet:

4. Does your child eat one or more meals a day with the family?
   - □ Never or rarely
   - □ Sometimes
   - □ Often
   - □ Almost always
   - □ N/A (such as for a breast-fed or a formula-fed infant)

5. Has your child seen his/her doctor since his/her discharge?
   - □ Yes
   - □ No

6. Has your child seen a dietitian since his/her discharge?
   - □ Yes, purpose of visit? ________________________________
   - □ No (if no, skip to question 8)

7. Where did he/she see the dietitian?
   - □ Hospital clinic
   - □ Private clinic
   - □ Home visit

8. Have you talked to another health professional about your child's diet or food intake?
   - □ Yes, what was the concern? ___________________________________
   - □ No

9. Has your child suffered from any complications since his hospital discharge 30-days ago?
   - □ Yes, please specify. ___________________________________
   - □ No

10. Has your child been re-hospitalized?
    - □ Yes, what was the reason? ___________________________________
    - □ No
